# Dual-view light-sheet imaging through a tilted glass interface using a deformable mirror

**DOI:** 10.1364/BOE.416737

**Published:** 2021-03-18

**Authors:** Nikita Vladimirov, Friedrich Preusser, Jan Wisniewski, Ziv Yaniv, Ravi Anand Desai, Andrew Woehler, Stephan Preibisch

**Affiliations:** 1 Berlin Institute for Medical Systems Biology (BIMSB), Max Delbrück Center for Molecular Medicine (MDC), Berlin, Germany; 2 Confocal Microscopy and Digital Imaging Facility, Experimental Immunology Branch, National Cancer Institute, National Institutes of Health, Bethesda, Maryland 20892, USA; 3 Office of Cyber Infrastructure and Computational Biology, National Institute of Allergy and Infectious Diseases, National Institutes of Health, Bethesda, Maryland 20894, USA; 4 Francis Crick Institute, Making Science and Technology Platform, London NW1 1AT, UK; 5 HHMI Janelia Research Campus, Ashburn, Virginia 20147, USA; 6 nikita.vladimirov@mdc-berlin.de; 7 stephan.preibisch@mdc-berlin.de

## Abstract

Light-sheet microscopy has become indispensable for imaging developing organisms, and imaging from multiple directions (views) is essential to improve its spatial resolution. We combine multi-view light-sheet microscopy with microfluidics using adaptive optics (deformable mirror) which corrects aberrations introduced by the 45^o^-tilted glass coverslip. The optimal shape of the deformable mirror is computed by an iterative algorithm that optimizes the point-spread function in two orthogonal views. Simultaneous correction in two optical arms is achieved via a knife-edge mirror that splits the excitation path and combines the detection paths. Our design allows multi-view light-sheet microscopy with microfluidic devices for precisely controlled experiments and high-content screening.

## 1. Introduction

Over the last decade, light-sheet fluorescence microscopy (LSFM, also known as selective-plane illumination microscopy, SPIM) has become one of the primary tools for imaging live developing organisms due to its low photo-toxicity, optical sectioning, isotropic resolution and high speed [1,2]. A conventional light-sheet microscope uses two objectives orthogonal to each other: one creates light-sheet excitation, while the other detects light emitted by the fluorescently labelled sample. The effective spatial resolution of LSFM can be improved with so-called multi-view imaging, in which orthogonal views from two or more objectives are computationally merged, resulting in isotropic resolution [3,4].

To minimize refractive index changes, the sample is usually embedded in agarose and suspended in aqueous imaging medium. However, this embedding method restricts the range of manipulations with the sample: fast application of drugs or chemical stimulants to the sample becomes difficult due to the presence of agarose barrier and large volume of imaging medium. Furthermore, due to the labor-intensive procedure of sample embedding and mounting, high-content screening becomes impractical.

Microfluidic chips permit tightly controlled experimental conditions, as the samples can occupy individual channels and be exposed to continuous flow, ensuring nutrient and chemical stimulant delivery, and simultaneous metabolic waste removal. Furthermore, microfluidic chips allow highly parallel experiments under nearly identical conditions. Conventional chips used in academic research consist of a PDMS (polydimethylsiloxane) polymer layer, in which three-dimensional structures (e.g. channels) are formed and then enclosed with a glass coverslip.

The presence of the glass coverslip is a major obstacle to the compatibility of LSFM with microfluidic chips. The orthogonality of objectives in a conventional light-sheet microscope requires that the glass coverslip is positioned at a 45^o^ angle to the objectives, which creates severe optical aberrations in both excitation and emission optical pathways, resulting in degradation of image quality. To address this problem, McGorty et al [5] corrected aberrations in the detection arm using adaptive optics (AO), but this approach did not generalize to multi-view imaging. Glaser et al [6] used a solid-immersion lens (SIL) that minimized aberrations in both excitation and detection arms, also in a single-view arrangement. Alternatively, several other designs [7–11] used one high-NA objective for both excitation and detection, at the cost of adding secondary and tertiary objectives used to re-image the tilted detection PSF onto the sensor.

In this work we present a dual-view AO selective plane illumination microscope (daoSPIM) design which allows high quality imaging of live organisms without additional custom devices in front of the coverslip or the presence of secondary and tertiary re-imaging objectives. The optical aberrations caused by the tilted coverglass of the microfluidic device are efficiently compensated by a deformable mirror. Moreover, due to the system’s symmetry, the single deformable mirror corrects aberrations in both optical arms simultaneously, thus simplifying the use of microfluidic devices for LSFM imaging in a cost-efficient way.

## 2. Results

**Fig. 1.**
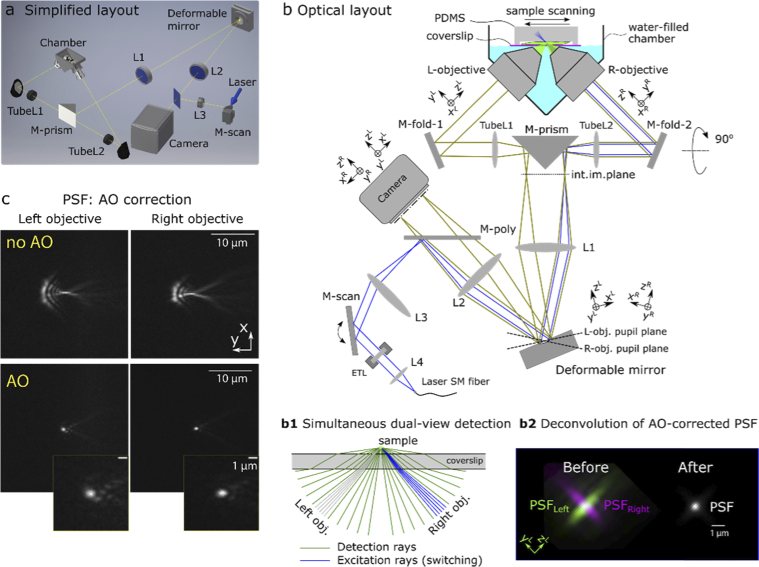
Microscope overview. (**a**) Simplified 3D model of the microscope layout. Scanning stage, mounts, and ETL module are omitted for clarity. (**b**) Optical layout with ray tracing: detection rays in green, excitation rays in blue. See **Visualization 1** for animation of laser scanning method. The plane of objectives and folding mirrors is orthogonal to the plane of the prism mirror, DM and camera. Note the transformations of local coordinates due to reflections, explained in the main text. Abbreviations: *TubeL1,2*, tube lenses; *M-fold*, folding mirror; *M-prism*, prism mirror; *L1,2,3,4,* achromatic lenses, *M-poly*, polychroic mirror, *M-scan*, scanning galvo mirror, ETL, electro-tunable lens; *SM*, single-mode fiber from laser head. (**b1**) Excitation can be directed into the left or right objective (gray or blue rays, respectively), while detection is performed by both objectives simultaneously. (**b2**) dual-view deconvolution of the AO-corrected PSF. (**c**) Fluorescent bead image in two orthogonal views, before DM correction (no AO) and after correction (AO).

The light sheet is generated by scanning a focused laser beam through one objective, and collecting fluorescence through two objectives, while the sample is mechanically scanned through the excitation plane. The microscope is arranged in an open-top configuration (**Fig. 1(a)**) for maximal user convenience, so that the coverslip-mounted sample is dipped into a water-filled chamber from the top (**Fig. 1(b)**).

Due to the high cost of the deformable mirror (DM), it is desirable to use a single DM for AO correction in both arms, which usually requires an optical switching mechanism that involves mechanical motion (e.g. flipping mirror). We avoided mechanical switching by adding a knife-edge prism mirror between the two arms (**Fig. 1(a,b)**, *M-prism*). We placed the deformable mirror at the intersection of the conjugate pupil planes of both objectives (**Fig. 1(b)**) to correct detection aberrations in both views simultaneously.

### 2.1 Excitation path

The light-sheet is generated by scanning the excitation laser beam with the galvo mirror *M-scan* (**Fig. 1(b)** blue rays). The laser beam starts in the joined path (laser out of *SM fiber -> L4 -> ETL -> M-scan -> L3 -> M-poly -> L2 -> DM -> L1*) and, depending on the galvo mirror voltage bias, reflects off the prism mirror *M-prism* into left or right arm. Thus, the galvo mirror *M-scan* serves two purposes: it switches the excitation between the arms, and scans the focused laser beam to create a light sheet plane in the active arm. An electro-tunable lens (ETL), placed before the galvo mirror, is used to adjust the light sheet axially.

### 2.2 Detection path

The fluorescence light emitted by the sample, collected by both objectives simultaneously (**Fig. 1(b)**, green rays), is combined side-by-side by the *M-prism*. It then reflects off the DM, and is focused by lens *L2* on the detection camera sensor (**Fig. 1(b)**, green rays). The images from both arms are thus acquired side by side during the same exposure. Thereafter, dual-view deconvolution combines the two AO-corrected views into one, with improved spatial resolution (**Fig. 1(b2)**). Due to symmetry of the optical system, the DM shape affects both views simultaneously and identically; thus, aberrations in both views can be corrected by the same DM command (**Fig. 1(c)**).

Through the paper we label the optical axes of objectives as **z**, the lateral axes of highest optical aberrations as **y**, and the other lateral axis (lowest aberrations) as **x**. Due to reflections from *M-fold1,2* (vertical plane in **Fig. 1(a)**) and *M-prism* (horizontal plane), the objective coordinates transform in such a way that their y-axes, orthogonal in sample/objective space, become collinear in DM and image space (**Fig. 1(b)**). At the same time, their x-axes, collinear in sample/objective space, become opposite in DM and image space. This makes possible correction of the aberrations simultaneously by the same DM command, if they are identical along y-axes and symmetric along x-axes, such as those imposed by a coverslip.

### 2.3 DM optimization algorithm

**Fig. 2.**
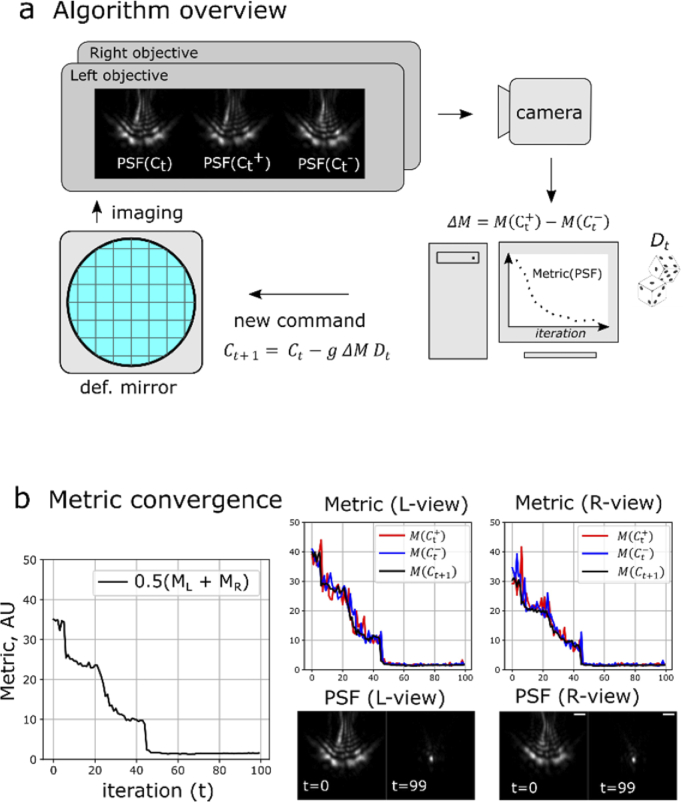
Algorithm for DM shape optimization. (a) Algorithm overview. At each iteration, three images are taken by the camera, and PSFs in each view are measured using metric *M*. (b) Total weighted metric convergence (left panel), and individual-view metric convergence (middle and right panels). See **Visualization 2** for PSF changes over iterations. Notation: $C_{t}$ is the DM command vector at time *t, g* is the gain, $D_{t}$ is the vector of random perturbations, $M(C_{t})$ is the metric of PSF image obtained after command $C_{t}$ was applied to the DM. Scalebar 2 µm.

In order to find the optimal DM shape for aberration correction, we adapted the stochastic parallel gradient descent (SPGD) algorithm [12], where we used dynamic gain control and an image-based metric that penalizes large-sized PSFs in both arms simultaneously (**Methods**). We found that the algorithm robustly and rapidly converged to a minimal PSF in both arms (**Fig. 2**): cross-section FWHM(x,y) mean ± std (0.44 ± 0.01 µm, 0.75 ± 0.01 µm, n=*10*) in the right arm, and (0.49 ± 0.05 µm, 0.74 ± 0.02 µm, *n=10*) in the left arm, measured with green fluorescent beads (diameter 0.17 µm, λ_em_ ∼ 515 nm). The measured PSF in diffraction-limited conditions (beads in agarose sheet, water, no coverslip) was (0.44 ± 0.003 µm, 0.49 ± 0.04 µm, *n*=3). The theoretical diffraction-limited FWHM is 0.39 µm for NA=0.8 objectives used. The difference between the no-coverslip PSF and the theoretically expected size can be explained by the physical bead size and a noticeable astigmatism from the 4% agarose layer used for bead mounting. The fact that AO-corrected PSF is elongated along Y axis is physically justified by the fact that a portion of NA in this direction is cropped by reflections off the coverslip [5] and the steepest phase gradient occurs along this axis at the pupil edge.

The DM optimization algorithm typically converged to a stable minimum in 50-60 iterations (2-3 minutes). The steep change of the metric (over 20x) was mitigated by the dynamic gain control, in which the gain increased inversely proportionally to the metric. This allowed small gain at the beginning of optimization (stability) and high gain at the end (sensitivity). Once the optimal DM shape was found, it could be used for multiple experiments, provided that nominal coverslip thickness remained the same and the optomechanical alignment of the microscope was stable.

### 2.4 Detection field of view

**Fig. 3.**
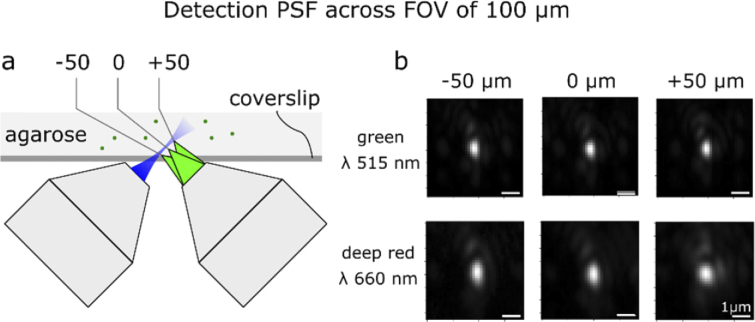
PSF measured using green and red fluorescent beads across 100 µm field of view (a). The DM shape was optimized using green beads. The PSF cross-sections are shown in (b). See Results for FWHM values.

After finding the optimal (green-channel, ex/em 488/515 nm) PSF at the center of camera’s field of view (FOV), we measured detection PSF across the FOV to determine the spatial range (isoplanatic patch) across which a single DM command can correct aberrations. We found that the isoplanatic patch spans about 100 µm along the Y axis (**Fig. 3(a,b)**), in which the green-channel PSF FWHM remains in the range 0.46-0.47 (x) 0.75-0.82 (y) µm: FWHM(x,y) = 0.47, 0.75 (y-pos. 0); 0.46, 0.82 µm (y-pos. -50 µm), 0.47, 0.77 (y-pos. +50 µm).

With the camera sensor split between two objectives and total system magnification 44.4x, this makes the useful detection FOV ca. 100 × 100 µm for each objective (with adjustment to FOV cropping due to the *M-prism* edge).

### 2.5 Multi-color detection

Because reflection changes the absolute optical path independently of the wavelength, the deformable-mirror based AO does not introduce additional chromatic aberrations beyond those already present due to refractive optical elements. Therefore, the DM shape optimized for the green-channel PSF (λ_em_ ∼ 515 nm) can be directly applied to the image PSF in deep red channel (λ_em_ ∼ 660 nm) (**Fig. 3(b)**). The deep red PSF size scaled approximately proportionally to the emission wavelength (660/515 = 1.28x) across the 100 µm FOV: measured FWHM was 0.59-0.87 (x) 1.01-1.09 (y) µm: FWHM(x,y) = 0.59, 1.03 (y-pos. 0); 0.59, 1.09 (y-pos. -50 µm), 0.87, 1.01 (y-pos. +50 µm). Thus, the detection system was close to achromatic, and the DM shape optimized for one color channel can be directly applied to other channels.

### 2.6 Dual-view PSF fusion

**Fig. 4.**
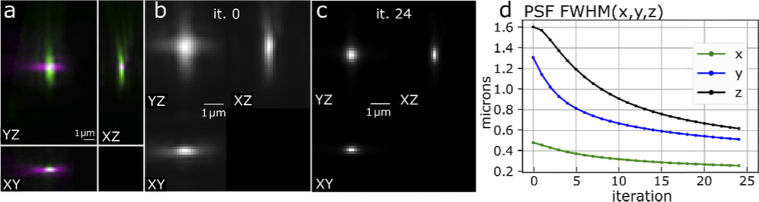
Dual-view PSF deconvolution, applied to AO-corrected PSFs. (**a**) Overlaid AO-corrected PSFs from the two views, acquired with light-sheet illumination. (**b**) Merged initial PSF, iteration 0. (**c**) Merged final PSF, iteration 24. (**d**) Changes of the merged PSF size (FWHM) during deconvolution.

PSFs from the two AO-corrected views were computationally combined into a single PSF with a multi-view deconvolution algorithm using an additive update scheme (**Methods**). This reduced effective 3-dimensional PSF size FWHM(x,y,z) from (0.52, 0.79, 2.82) down to (0.26, 0.51, 0.62) µm, thus improving the PSF by factors (2.0, 1.5, 4.5) in (x,y,z) respectively (**Fig. 4**). Note that X axis has smallest optical aberrations, Y axis has the largest aberrations, and Z axis in one PSF corresponds to Y axis of the other PSF after the two views are brought into the same coordinate system.

### 2.7 Light-sheet profiles

**Fig. 5.**
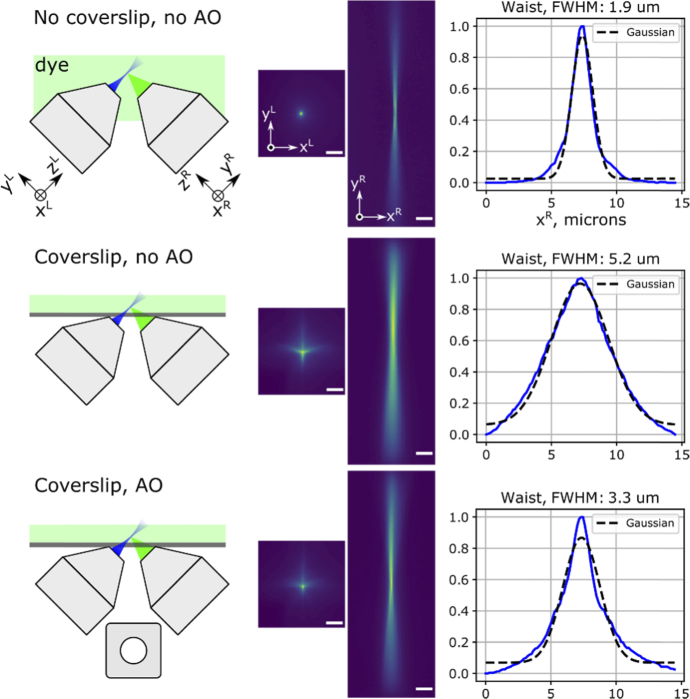
Light-sheet excitation beam properties. Image of the laser beam (NA 0.16) in fluorescein solution: no coverslip, with flat DM; after the beam passed through the coverslip without AO (flat DM); with AO (DM shape optimized for detection PSF); Excitation 488 nm, emission ∼515 nm. Scalebar 20 µm.

Because of the achromatic nature of deformable mirror correction, the excitation light receives a phase shift identical in amplitude to detection light, but opposite in sign, and across a smaller pupil area (corresponding to light-sheet NA compared to detection NA). We thus expect that the detection-optimized DM shape also makes pre-shaping of the excitation laser, so that after passing through the coverslip it becomes closer to a diffraction-limited Gaussian beam. Indeed, we found that green-channel detection AO significantly improved the laser beam profile (**Fig. 5**). We visualized beam profiles for 488 nm and 561 nm excitation lasers as they illuminated green or red dye (fluorescein or rhodamine B, respectively) between the coverslips. We found that the AO-corrected beam waist was about 3.2 µm for both wavelengths, compared to coverslip-free 1.9 µm, and the non-AO-corrected coverslip beam waist was 5.2 µm. Thus, green-channel detection AO correction provided satisfactory improvement for excitation beam profiles in both 488 and 561 nm excitation channels, albeit the beam waist was thicker than in the no-coverslip case (**Table 1**).

**Table 1.** Beam profiles in fluorescent dyes, with and without AO, compared to no-coverslip conditions.

Beam parameter	No coverslip, no AO		Coverslip and AO		Coverslip, no AO	
Excitation wl, nm	488	561	488	561	488	561
	
Beam waist^*a*^, µm	1.9	1.9	3.3	3.1	5.2	5.2
Beam length^*b*^, µm	28.2	21.4	60.8	44.9	90.4	79.4

^*a*^ Measurement errors ±0.1 µm (std).

^*b*^ Beam length = 2x Rayleigh range.

### 2.8 Imaging of C. elegans in a microfluidic chip

We designed and manufactured microfluidic chips for imaging physically restrained *C.elegans* nematodes in tapered PDMS channels covalently bound to a high-precision #1.5H coverslip glass. The channels have a tip opening of 3 µm that prevents the nematodes from escaping. Nematodes with panneuronally expressing GCAMP6 (*AML32* line, dauer stage), were anesthetized and kept in the channels by applying mild static pressure.

**Fig. 6.**
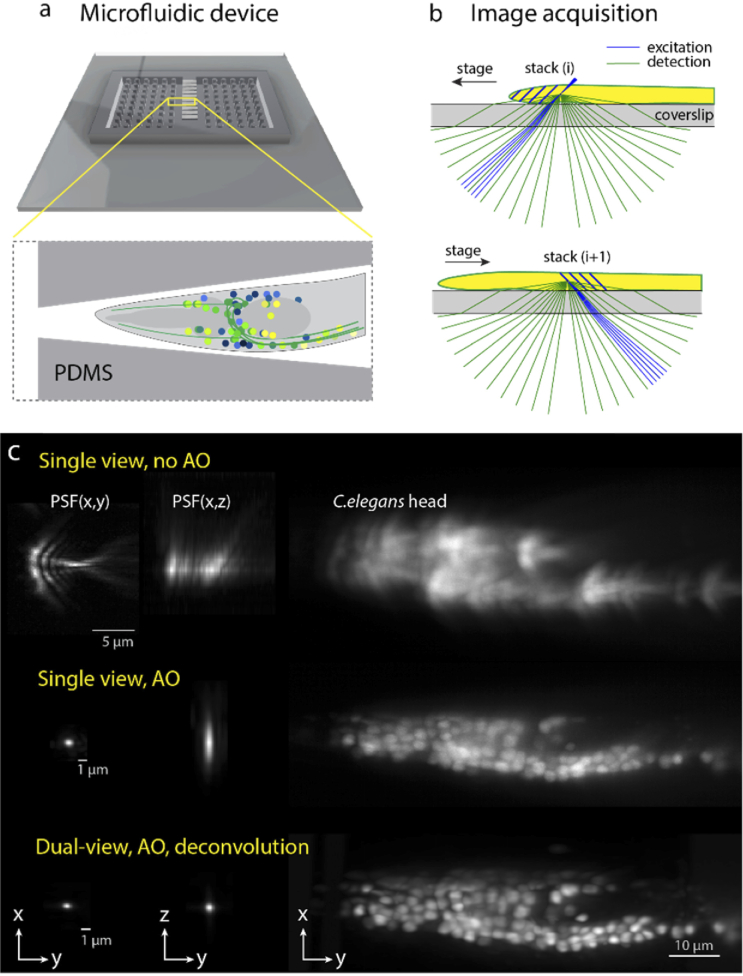
Dual-view imaging of *C.elegans* in microfluidic chip. (**a**) overview of microfluidic device on glass coverslip. (**b**) Stack acquisition scheme: right view is acquired when scanning the sample on one direction, left view when scanning in the opposite direction. (**c**) Point spread functions and single-view stack MIP of *C.elegans* head neurons acquired through the coverslip without AO (top row), with AO (middle row), and dual-view acquisition with AO, deconvolved and merged (bottom row).

After optimizing the DM shape using fluorescent beads, we imaged the head of an immobilized animal through the coverslip, along with individual fluorescent beads mounted in agarose to acquire PSFs for dual-view deconvolution (**Fig. 6**). Imaging with AO correction significantly improved image quality in each view over the image without AO, making individual neurons distinguishable. Computational merging of the left and right views by dual-view deconvolution further improved the image resolution, as expected from the nearly isotropic reconstructed PSF.

## 3. Methods

### 3.1 Optomechanical design

The microscope components, their approximate cost and spatial arrangement are described in the daoSPIM github repository: https://github.com/nvladimus/daoSPIM/wiki/overview.

The microscope has two water-dipping objectives (40X Nikon CFI APO NIR Objective, 0.80 NA, 3.5 mm WD) fitted in a custom 3D printed chamber using custom-made silicone rubber O-rings. The objectives were fixed at 90^o^ angles to each other on manual Z-axis translation mounts (Thorlabs SM1Z) to allow fine axial alignment. The microfluidic chip on a 22 × 22mm coverslip glass (#1.5H, VWR International, #630-2186) is held by small magnets in a sample holder and can be translated horizontally by a motorized stage (ASI FTP flat-top stage, scan-optimized, 4TPI), and vertically by a manual translation stage (Newport M-461-X-M) with a small dove rail for quick sample holder release. The sample holder was custom-designed and manufactured from stainless steel (Protolabs). The 3D models of chamber, O-rings, and sample holder are available at the project repository.

The folding mirrors *M-fold1,2* (Thorlabs BBE1-E02) are mounted on 45° mounts (Thorlabs H45E1) on top of kinematic mounts (Thorlabs KM100) and post assemblies for fine and crude adjustment, respectively.

**Fig. 7.**
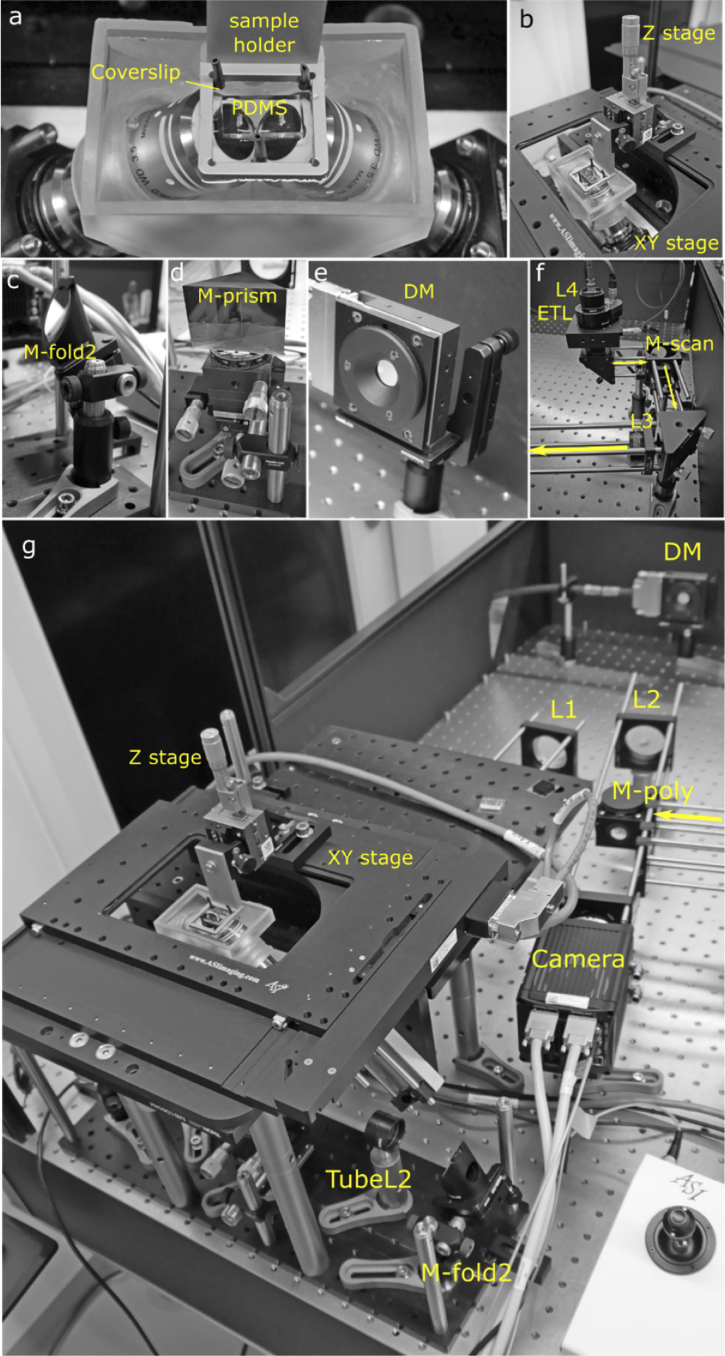
Optomechanical implementation. (**a**) Water-filled chamber with two objectives in open-top configuration. The microfluidic device is fixed inside the sample holder. (**b**) The sample holder is attached to motorized horizontal stage and manual vertical stage. (**c**) The folding mirror assembly. (**d**) The M-prism is mounted on the XYθ mini-stage. (**e**) The DM is fixed on a kinematic prism mount. (**f**) Laser scanning assembly, with yellow arrows showing laser propagation direction. (**g**) System overview, with the laser coming from the laser scanning assembly (yellow arrow).

The knife-edge mirror *M-prism* (Edmund Optics #47-006) was glued to an XYθ stage assembly (Standa 7R7E, 7T273-10T). The DM was fixed on a kinematic prism mount (Thorlabs KM200PM/M) and a custom adapter. The camera (Hamamatsu Orca Flash 4.3), polychroic mirror (Chroma ZT405/488/561/640rpcv2-UF2) and lens *L2* (Thorlabs AC508-400-A-ML) were mounted in a 60-mm cage assembly. Details of the optomechanical implementation are shown in **Fig. 7**.

We used a continuous faceplate deformable mirror (Mirao52e, Imagine Optic), which has 52 electromagnetic actuators over an aperture of 15 mm. The back focal planes of both objectives were magnified and relayed on the DM faceplate position via lenses TubeL1/TubeL2 and lens L1 and beam combiner *M-prism*.

### 3.2 System alignment

Our microscope reuses several optical elements (M-prism, DM, L1, L2 and individual arms) for up to 4 purposes: imaging through the left and right objective, and light sheet generation through them. Because of this, it requires careful alignment in the remaining degrees of freedom, so that aligning for one function does not misalign the other three. We solved the problem using the following framework:

1. Initial mechanical alignment is done using a screw-in laser pointer guide and alignment targets (Thorlabs) placed on optical cages and lens mounts (L1, L2, TubeL1, TubeL2, objectives).
2. Optical alignment is based on a camera image with overlays to indicate the centerpoint and centerlines.
  Initial optical alignment is performed by making aperture centers concentric with the image center, starting with those which are proximal to the camera, and using screw-in frosted-glass alignment disks (Thorlabs).
3. Temporary imaging lenses are placed instead of L2 to allow imaging of system apertures at various positions. The DM aperture is imaged by placing achromatic lens L2’ of half the focal distance of L2 (f=400/2 = 200mm) instead of L2. The pupil aperture of each objective is imaged on top of the DM aperture, by illuminating the sample space with diffuse light and closing the other objective path.
4. The detection path is aligned before the excitation.
5. The alignment is finalized by imaging the beam profile of a 488nm laser in fluorescein bath. The beam images (cross-sectional and longtitudinal) should be in FOV center of corresponding views.

More details at https://github.com/nvladimus/daoSPIM/wiki/Alignment.

### 3.3 Optimization procedure

We imaged green fluorescent beads mounted between two coverslips in 4% agarose and distilled water. The region of interest (ROI) in each arm was selected to contain a single bead (the same bead in two orthogonal views), and was typically 100 × 100 px^2^ in size. The light-sheet laser was defocused to provide quasi-wide-field excitation, in order to avoid artefacts related to the laser beam walking off the bead, since both detection and excitation paths are controlled by the same deformable mirror. A Python notebook with example optimization code is provided at the github repository https://github.com/nvladimus/daoSPIM.

### 3.4 Optimization metric

For DM shape optimization we adapted SPGD, **s**tochastic **p**arallel **g**radient **d**escent algorithm [12] and minimized the following image metric $M_{view}=\int I(r)r^{2}d^{2}r$.

Here *I(r)* is the PSF image intensity (in left/right view) normalized to be within [0,1], *r* is the radius from PSF center. The SPGD should not be confused with SGD (stochastic gradient descent) commonly used in machine learning.

This metric choice was inspired by astronomy applications [13]: it penalizes large-sized PSFs due to rapidly increasing *r^2^* term from the PSF center. The PSF center position was determined by thresholding the brightest pixels in the image (99% percentile) and computing their center of mass. After the main optimization, fine-tuning was sometimes necessary to further minimize FWHM(x,y) dimensions of the nearly-optimal PSF by another 15-20%. This was done by fitting PSF with 2D Gaussian profile and computing its FWHM(x,y).

All optimizations were performed on the metric averaged between the two arms,

$$M=0.5(M_{L}+M_{R})$$

Since there are individual differences between the two optical arms, this metric balanced between them. However, the algorithm also performed well when one arm was given a preference (weights 0.75:0.25).

### 3.5 Optimization: initial conditions

The optimization had to start from relatively flat DM, so that the initial PSF is not too distorted due to initial mirror shape. We initialized DM from a factory-calibrated “flat” command or from an all-zero command (non-flat DM shape), and found the latter gave a more reproducible final optimized state, presumably because there is less initial bias compared to the factory-provided flat command. Initial gain was set to 0.03, because values in the range of (0.01 to 0.05) gave the most stable convergence.

### 3.6 Optimization: iterations

At each iteration *t*, the current DM command $C_{t}={\left(c_{1},\ldots ,c_{N}\right)}_{t}$ (*N*=52 is the number of DM actuators) was perturbed in two opposite directions by a random vector $D_{rand}=(d_{1},\ldots ,d_{N}):C_{t}^{+}=C_{t}+D_{rand};C_{t}^{-}=C_{t}-D_{rand}.$

The components of $D_{rand}$ have randomly chosen signs and fixed amplitudes $abs(d_{i})=0.002V\;(i=1,..,N)$. The perturbation amplitude of 0.002 V was empirically found to provide sufficient difference ΔM in metric between resulting images, but small enough to keep the algorithm stable. The new command was computed from the difference in cost function *M* between these two perturbations [12]:

$$C_{t+1}=C_{t}-g\;\Delta M\;D_{t}$$

Here *g* is the gain parameter, $\Delta M=M(C_{t}^{+})-M(C_{t}^{-})$ is the increment in cost function if commands C_t_^+^ and C_t_^-^ are applied to the DM. The gain *g* is a free parameter that critically affects the algorithm performance. Lower gain ensures stable convergence but slow speed of the algorithm, which is undesired due to increased sample exposure. High gain provides fast convergence but the algorithm can become unstable.

### 3.7 Optimization: dynamic gain control

In our case, the metric *M* changed considerably (by more than 20x) due to the highly distorted initial PSF image. To achieve both stable and fast convergence we made the gain adaptive [14], so that it is scaled based on initial and current metric values:

$$g(t)=g_{0}\frac{M_{0}}{M(t)}$$

Here *g_0_* is the initial gain (0.03), $M_{0}=M(C_{0})$ is the initial metric value with DM command $C_{0}=(0,0,..0)$, and $M(t)=M(C_{t})$ is the metric at iteration *t* with DM command $C_{t}$. This dynamic gain adjustment ensured that the algorithm becomes more sensitive to very small changes of the cost function *M* as the bead image approaches the diffraction-limited PSF size, which is only 3-5 pixels wide.

Due to the stochastic nature of the algorithm and presence of noise, variations in convergence and final state (PSF shape) are inevitable. However, the algorithm failed to converge only in marginal cases: when more than one bead was present in the optimization ROI, or when the bead image was too dim (SNR ratio less than 10). In all successful cases, variations in the optimized PSF shape concerned only the shape of (small) side lobes, and the algorithm converged in less than 100 iterations (5 min). Variations in the optimized DM shape can also come from the fact that the same PSF can be achieved by multiple commands due to invariance of PSF (x,y) translation that corresponds to DM tip and tilt, respectively.

### 3.8 Optimization: regularization

In order to employ the left-right symmetry of the optical system (X^L^ vs X^R^ at DM in Fig.1b) and the expected left-right symmetry of the DM shape, we added a regularization step before updating DM command after each iteration, which averaged actuator commands with symmetrical positions relative to the DM middle line:

$$\begin{array}{c} {C^{reg}}_{t+1}(ID_{left})=0.75C_{t+1}(ID_{left})+0.25C_{t+1}(ID_{right})\\ {C^{reg}}_{t+1}(ID_{right})=0.75C_{t+1}(ID_{right})+0.25C_{t+1}(ID_{left}) \end{array}$$

where $ID_{left}$ and $ID_{right}$ are the corresponding left and right actuator identification numbers (1,..,52).

### 3.9 Optical simulations

**Fig. 8.**
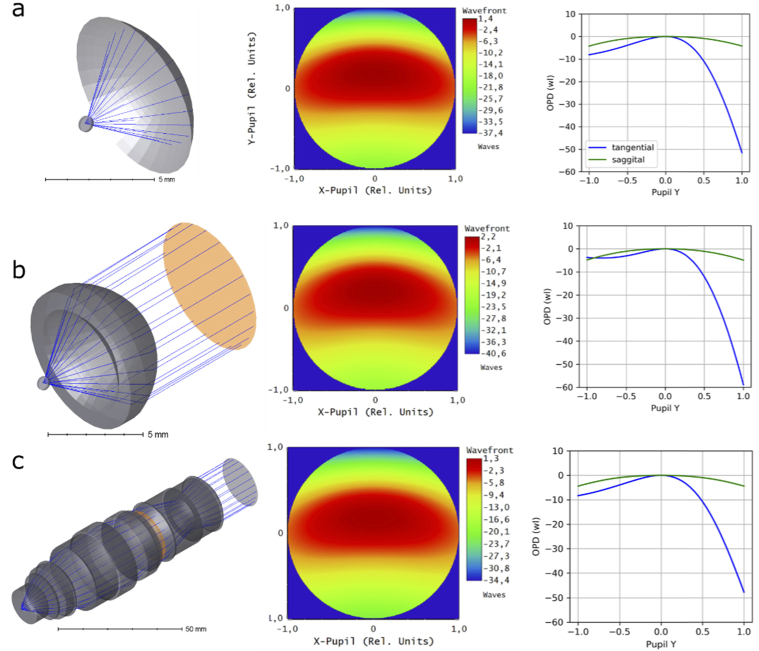
Ray tracing models and their predictions. (a) Model 1, coverslip tilted in water and spherical reference surface; (b) model 2, single aspheric lens in water; (c) model 3, Nikon 16x/0.8 water-dipping objective. Pupil planes are shown in orange. Second and thirds columns show simulated wavefronts relative to centroid ray (with y-tilt subtracted) and optical path difference to chief ray (with y-tilt preserved), respectively. OPD is shown in wavelength units (λ=520 nm).

To simulate the system wavefront at the pupil plane of objective accurately, one needs a model of the particular objective (in our case 40X Nikon CFI APO NIR Objective, 0.80 NA, 3.5 mm WD). Since the exact design is proprietary, we used three simplified models to assess the system wavefront (**Fig. 8**). All models were made in Zemax.

Model 1: a reference sphere in water. In the simplest approximation, we only inserted a tilted coverslip in water and plotted the relative phase map of rays at the reference spherical surface positioned 5 mm (focal distance of the objective) away from the point source. No other optical elements were used in this model, which minimized assumptions made. Coverslip thickness was 170 µm, tilt 45^o^, glass type Schott.

Model 2: a single aspheric lens objective. In this case, we modelled a 40x/0.8NA water-dipping objective by using a single lens of high-RI material (RI=2.41, similar to diamond), with spherical front surface (no refraction) and hyperbolic second surface, where all refraction occurs. This is a reasonable model of an objective for a monochromatic point source positioned in focus on the optical axis.

Model 3: a 16x/0.8NA objective model. We also used a detailed model of 16x/0.8NA Nikon water-dipping objective (kindly provided by D. Flickinger).

Despite the different assumptions of each model, their predicted aberrations agree. The main aberrations are: vertical tilt, vertical astigmatism, defocus, vertical coma and vertical trefoil (listed in decreasing amplitude order), see **Table 2**.

**Table 2.** System wavefront RMS according to the models.^*a*^

Coefficient	Model 1 no lenses	Model 2 diamond lens	Model 3 Nikon 16x/0.8
Global y-tilt of the wavefront	-14.2	-18.3	-13.3
PV (to centroid) after y-tilt subtraction	38.8	42.8	35.7
RMS after y-tilt subtraction	7.6	7.9	7.1
Z3 (remaining y-tilt)	-7.1	-9.1	-6.7
Z4 (defocus)	-4.8	-5.1	-4.7
Z5 (o. astigmatism)	0	0	0
Z6 (v. astigmatism)	5.3	5.4	5.0
Z7 (v. coma)	-2.1	-2.7	-1.9
Z8 (h. coma)	0.0	0.0	0.0
Z9 (v. trefoil)	0.8	0.9	0.7
Z10 (o. trefoil)	0	0	0
Z11 (spherical)	0	0.04	0.06

^*a*^ All units are in wavelength (λ=520 nm), the point source is on the optical axis.

### 3.10 Detection PSF measurement

We used green (ex. 505, em. 515 nm) and deep red (630/660 nm) fluorescent beads 0.17 µm in diameter, from Invitrogen TetraSpeck Fluorescent Microspheres Sampler Kit. The beads were quickly mixed in 4% low-melting point agarose (Genaxxon Bioscience) at 70^o^ C and sandwiched between two coverslips. The coverslips (#1.5H, VWR International, #630-2186) were sealed with dental glue (picodent twinsil speed).

### 3.11 Excitation laser beam profiling

For profiling 488 nm laser beam shape, we mixed fluorescein chloride (Sigma-Aldrich Chemie) with distilled water and sealed the solution between two coverslips with glue (picodent twinsil speed) and spacers of about 100 µm thickness. For 561 nm laser beam profiling, we used Rhodamine B red (ChemCruz) in distilled water.

### 3.12 Hardware synchronization

In order to capture optical sections of a sample with high speed and precision, the system requires synchronization between illumination, camera, and stage motion with an accuracy of at least 0.1 ms. Our system was driven by scanning XY stage (ASI) moving along the scanning direction with TTL pulses fired every *ds* µm (0.5 to 2.8 µm, depending on the sample). The pulses emitted by stage triggered camera readout events (Hamamatsu SYNCREADOUT mode). The camera fires pulses that indicate the global exposure period, which are captured by a digital counter task of an NI DAQmx board. The latter generates re-triggerable analog waveforms for galvo mirror swipe and laser ON commands.

### 3.13 Image acquisition

The sample was moved at a constant speed through the light sheet generated by scanning a Gaussian beam with the galvo mirror. Such light-sheet illumination mode (in contrast to stationary light-sheet generated by a cylindrical lens) ensures that there is no motion blur, since the illumination is effectively stroboscopic: laser swipe time across the sample plane is 2 ms, the dwell time at any point is less than 0.1 ms, while the time interval between consecutive planes is 5-10 ms (the camera exposure time).

The image stacks were streamed into an HDF5 file along with an un-shearing affine transformation matrix that converted scanning stage intervals *ds* between successive images into corresponding *dz* intervals between planes ($dz=ds/\sqrt{2}$ µm). Thus, unsheared stacks could be visualized directly in BigStitcher without any post-processing.

### 3.14 Laser switching between the arms

In order to switch the laser direction between the arms, one needs to add or subtract a voltage bias to/from the galvo-driving analog command after every *N*(images per stack) times. It was problematic to implement a re-triggerable analog task in PyDAQmx that meets this condition, so we added a custom PCB board (based on Teensy 2.0 MCU) that counts the camera exposure pulses and adds the left or right arm voltage bias to the galvo-driving signal in real time. The PCB board design is available at the daoSPIM github repository.

### 3.15 Control software

The control software was written in Python and is available at the github repository https://github.com/nvladimus/daoSPIM. In short, we wrote modular control modules for individual devices (camera, DM, light-sheet generator, scanning stage, ETL) which can be used as stand-alone programs. A top-level code (Python PyQt5) combined them into a specific workflow program. The control software saved image stacks directly into HDF5 format using *npy2bdv* library [15]. The HDF5 format was chosen for compatibility with Fiji BigDataViewer/BigStitcher and Imaris software [16,17].

### 3.16 Registration between left and right arms

Stacks taken by both objectives were unsheared via affine transformation and resampled at the (x,y) resolution (0.14625 µm/px) via linear interpolation. Stacks taken by the right objective were transformed into the coordinate system of the left objective (transformations: inversion of X and Y axis signs, followed by rotation around OX axis by 90^o^). Once in the common coordinate system, both stacks were rotated +45^o^ to fit a rectangular box with minimum background present. Registration pipeline included intensity-based center-of-mass initial registration, intensity-based rigid registration, and iterative closest-point (ICP) method using points-of-interest (neurons).

### 3.17 Dual-view deconvolution

In order to compute fused image stack from the two views, we used an iterative dual-view Richardson-Lucy deconvolution [3,18–20] with experimentally measured PSF for each view.

For the registration and deconvolution we used BigStitcher [17] and simpleITK [21,22] software. Data analysis was performed using python *numpy* and *matplotlib* libraries [23,24].

### 3.18 Microfluidic device fabrication

The design consists of an inlet and outlet area, separated by 1.5mm long parallel, tapered channels to physically restrain nematodes. Standard microfabrication techniques were used to fabricate the microfluidic device [25]. Briefly, chip geometries were custom designed in CLEWIN software (WieWeb) and projected on SU-8 wafers using a MicroWriter ML3 (Durham Magneto Optics) optical lithography machine. Prior to photolithography, wafers were coated with SU-8 2010 (MicroChem) at a height of 15 µm (spin 10s - 500rpm, 100rpm/s acceleration; 30s - 1500 rpm, 30rpm/s). After exposing the wafer and baking according to SU-8 manufacturer’s instructions, wafers were developed by washing with PGMEA and isopropanol followed by a hardbake at 200°C for 20min. Master molds were used as negatives for polydimethylsiloxane (PDMS) replication. After applying PDMS (Sylgard 184, 1:10 ratio of curing agent and base), molds were baked at 110°C for 15 mins. PDMS devices were permanently bonded to #1.5 coverslips cleaned by a 30 second exposure to oxygen plasma. Bonded chips were baked on a hot plate at 75°C for one hour.

### 3.19 Worm handling

AML-32 nematodes expressing GCaMP6s in all neurons [26] were used for imaging. Dauer worms were extracted from starved plates that have been at 25°C for at least 5 days. Briefly, nematodes were washed off starved plates and washed twice with M9, before adding 1% SDS solution (dissolved in M9) and incubation for 30 mins at room temperature on a rotating wheel. Dauers were collected in M9 containing 1 mM tetramisol and loaded on microfluidic chips with the help of a 1ml syringe attached to a small piece of polytetrafluorethylene (PTFE) tubing.

## 4. Discussion

Our AO microscope design demonstrates the possibility of dual-view light-sheet imaging through a 45^o^ tilted coverglass interface that separates objectives from the living sample. We designed our prototype system with the requirements of using off-the-shelf water-dipping objectives (Nikon 0.8/40x) and aqueous sample of extended lateral dimensions (up to several mm).

In order to correct the extreme aberrations caused by the 45^o^ tilted glass coverslip, we employ a sensorless AO approach for PSF optimization with dynamic gain control. The latter speeds up convergence and ensures that the algorithm is not stuck in local minima. The algorithm currently optimizes PSF from a single fluorescent bead in the field of view. In the future, optimization of extended sources (biological or synthetic fluorescent samples) would be preferred for optimization of the detection PSF across the desired field of view. In principle, the use of AO is optional, since the aberrations due to the coverslip glass remain constant. Thus, the DM can be replaced by a fixed mirror with suitable surface curvature. However, the DM has additional ability to compensate for errors in alignment and variations in the coverslip thickness and flatness.

Although a smaller tilt angle (e.g. 40^o^) generates less aberrations and allows for a better PSF correction [5], we decided to use a 45^o^ tilt in order to achieve symmetry between the two arms, necessary for multi-view imaging. We also dispensed with the pre-compensating cylindrical lens. Performing correction with a reflective element (DM) does not introduce chromatic aberrations and allows using it for multiple fluorophores and excitation lasers for simultaneous multi-color imaging. The measured thickness of the excitation laser beam is further from the diffraction limit than the corresponding detection PSF, which requires further study.

The problem of open-top imaging through a 45^o^ tilted glass/polymer window was recently addressed by [6] with a solid immersion lens, refractive index matching and multi-immersion objective design. Our method provides a more aberrated PSF in each view and a smaller field of view, but higher magnification, higher spatial resolution and it is compatible with live, dual-view imaging. The two methods solve the tilted glass problem in different application niches, and therefore complement each other. Besides the demonstrated compatibility with microfluidics, our design opens possibilities of dual-view light-sheet imaging of live samples in glass-bottom dishes and multi-well plates.

Another approach to the problem of light-sheet imaging through glass uses a single objective for both excitation and detection [7–10]. Recent implementations achieve spatial resolution that matches high-NA spinning disk confocal imaging [8], and multi-view imaging via beam inversion by two additional galvo mirrors [11]. The latter, however, has non-orthogonal PSFs from the two views, which is likely to reduce the improvement of z-resolution from multi-view fusion. These systems require samples which have refractive index close to the imaging medium. Our AO approach has additional flexibility to correct some sample-induced aberrations: variations of RI which are stratified along the plane of the coverslip (e.g. thick live samples with skin, cuticle, or layers of tissues, either static or changing over time) could be treated the same way as aberrations caused by the coverslip, so they will be corrected by our system in both views simultaneously.

In our experiments, fluorescence collected by the objective that generates the light-sheet was usually discarded, because these images contain a max-projection of the sample along the illumination axis, which is uncommon in light-sheet microscopy. We used this view only for visualization of the laser beam cross-section (Fig. 5). However, this view can be used for other purposes: 3D tracking of particles, or simultaneous light-sheet imaging in a second color channel. The latter can be achieved, for example, by generating a light sheet alternatively in 488 nm, right arm, and 561nm, left arm, during a single camera exposure, and splitting the dual-view images by color using a second camera or an image splitter.

The optical symmetry of our system provides several unique properties that are relevant outside of these particular applications. Images from the two objectives are spatially separated on the camera chip, while their conjugate (Fourier) planes occupy the same spatial region (where we place the DM), and their phases differ only by tilt. This allows simultaneous correction of aberrations, or generation of custom excitation beams, in two arms using a single AO element.

Finally, the knife-edge mirror beam splitter we propose allows sharing camera, laser, and galvo scanner between the two arms, thus significantly reducing the cost of multi-view light-sheet microscopes.
